# Autologous Mesenchymal Stem Cell Transplantation in Multiple Sclerosis: A Meta-Analysis

**DOI:** 10.1155/2019/8536785

**Published:** 2019-12-23

**Authors:** Yang Zhou, Xin Zhang, Hang Xue, Lingling Liu, Jie Zhu, Tao Jin

**Affiliations:** ^1^Department of Neurology and Neuroscience Center, The First Hospital of Jilin University, Changchun, China; ^2^Department of Neurotraumatic Surgery, The First Hospital of Jilin University, Changchun, China; ^3^Department of Neurobiology, Care Sciences and Society, Karolinska Institute, Stockholm, Sweden

## Abstract

Multiple sclerosis (MS) is considered to be a central nervous system (CNS) chronic inflammatory demyelinating disease, affecting more than 2 million individuals worldwide. In this meta-analysis, we aimed to assess the safety and efficacy of autologous mesenchymal stem cells (aMSCs) in treating MS patients. The PubMed, Embase, Cochrane, Web of Science, and Clinical Trial databases were searched in September 2019. The analysis was conducted for three endpoints: transplant-related mortality (TRM), rate of disease progression, and no evidence of disease activity (NEDA) status. RevMan and the metaprop command of the meta package in R was used in assessing the efficacy and safety of aMSCs. Subgroup analyses were performed for exploration of heterogeneity regarding outcomes. Nine studies comprising 133 patients were included in the meta-analysis. The pooled estimate of TRM was 0% (95% confidence interval (CI) 0%–0.3%). The rate of progression was 16% at 6 months (95% CI 10%–27%) and 35% at 1 year (95% CI 27%–46%). Lower 6-month and 1-year progression rates were significantly associated with intrathecal injection (*p* = 0.02; *p* = 0.003). The pooled proportion of NEDA patients at 6 months was 72% (95% CI 58%–89%) and at 1 year was 62% (95% CI 42%–81%). Cell transplantation with aMSCs in MS patients is safe, with the largest benefit profile obtained in patients with aMSCs intrathecal injection.

## 1. Introduction

Multiple sclerosis (MS) is a chronic inflammatory demyelinating disease in the central nervous system (CNS), leading to demyelination, neurodegeneration, and gliosis [1]. At present, MS affects approximately 2,500,000 people worldwide, especially young adults, which presents a heavy burden for families and society [1, 2]. Although most effective disease-modifying therapies (DMTs) improve cognitive and physical disability in MS patients, there has been limited efficacy in these treatments and they are often related with side effects [1, 3]. Therefore, we need to develop a more effective treatment for MS patients.

Mesenchymal stem cells (MSCs) are derived from bone marrow (BM) and other tissues, including fat, muscle, umbilical cord blood (UCB), dermis, and dental pulp [4, 5]. MSCs have been used therapeutically in over 700 clinical trials which include heart diseases, stroke, and autoimmune diseases (multiple sclerosis, graft-versus-host disease, systemic lupus erythematosus, and so on) [5, 6]. In addition to their multipotential abilities, MSCs have also displayed immunoregulatory and neuroprotective properties [3, 4, 7]. Related studies on MSCs using the experimental autoimmune encephalomyelitis (EAE), an animal model for MS, have shown that increased numbers of regulatory T cells (Tregs), decreased demyelination, and inflammation [6]. MSCs also modify microglia cells by increasing the M2 and decreasing the M1 phenotype, exerting a protective role for MS [8]. Currently, the meta-analysis of autologous hematopoietic stem cell transplantation (aHSCT) has been evaluated in treating MS patients [9].

Therefore, the aims of this meta-analysis were to evaluate the efficacy and safety of MSCs by systematically collecting and summarizing all the evidence published about the outcomes including disease activity, Expanded Disability Status Scale (EDSS), and adverse events.

## 2. Materials and Methods

### 2.1. Search Strategy

We systematically searched all the published studies reporting aMSCs for MS on PubMed, Embase, Web of Science, Cochrane, and Clinical Trials (published up to September 25, 2019). Our search strategies including free words and MeSH terms (multiple sclerosis [MeSH] and transplantation [MeSH] and patient [MeSH]): (a) “mesenchymal stem cells” or “MSCs”, and (b) “multiple sclerosis” or “MS”, (c) patient. In addition, other included studies were collected manually from references of eligible studies or other articles related to this topic. The abstracts were examined independently by two authors (Y Zhou and X Zhang).

### 2.2. Selection Criteria

Inclusion criteria for this meta-analysis were as follows: any study (a) on MS; (b) on patients receiving aMSC transplantation; (c) including data on efficacy of aMSC transplantation; (d) reporting mortality and clinical follow-up; (e) including more than 5 patients; and (f) published in English.

The exclusion criteria were as follows: the study (a) did not meet the inclusion criteria; (b) was an editorial, review, case report, or abstract, or was from a clinical conference, comments, or congresses; and (c) involved nonhuman studies.

### 2.3. Data Extraction

The extracted data from eligible studies were as follows: (a) identity: authors, years, number of included patients; (b) baseline characteristics of patients (age, EDSS, proportion of patients with SPMS, disease duration, and relapses in the previous year); (c) treatments: transplantation methods, cell doses, follow-up period; and (d) outcomes: EDSS, mortality, and disease activity. The data from included articles were independently extracted and processed by two authors (Y Zhou and X Zhang). Any disagreement between the two authors was settled by consultation with a third author.

### 2.4. Types of Outcome Measures

The following endpoints were used to assess the safety and efficacy of aMSC on transplantation MS patients. The transplant-related mortality (TRM) was defined as death within 100 days of aMSCs transplantation, and the data of overall mortality (OM) was derived from the entire follow-up of all included studies. Progression events were defined as increasing 1 point (baseline EDSS ≤ 5.5) or 0.5 points (baseline EDSS > 5.5), and the EDSS score was, respectively, assessed at 6 or 12 months. No evidence of disease activity (NEDA) in MS patients was defined as without any disability progression, clinical relapse, or new MRI lesion (T2 or gadolinium-enhancing) over a limited period.

### 2.5. Statistical Analysis

The meta-analysis was completed using RevMan5.3 (The Cochrane Community, London, United Kingdom) and the metaprop command of the meta package in R to assess the safety (the transplant-related mortality) and efficacy (progression rate and the proportion of no evidence of disease activity). We used odds ratio (OR) and related 95% CI to analyze the data and log transformation to calculate pooled proportions under the fixed and random effects model. The chi-squared value test and inconsistency index statistic (*I*^2^) were used to evaluate statistical heterogeneity across each study. A value of *p* < 0.1 or I^2^ > 50% was indicated significant heterogeneity, and then the related data was analyzed with the random effects model. Otherwise, we used the fixed effects model. Subgroup analyses used the chi-squared test to analyze the subgroup data, with *p* < 0.05 indicating statistical significance.

### 2.6. Standard Protocol Approvals, Registrations, and Patient Consents

Written informed consent was acquired from all patients, and original data was approved by the local ethics committees. Any identifiable patient data were not found in this meta-analysis.

## 3. Results

### 3.1. Study Screen

**Figure 1:**
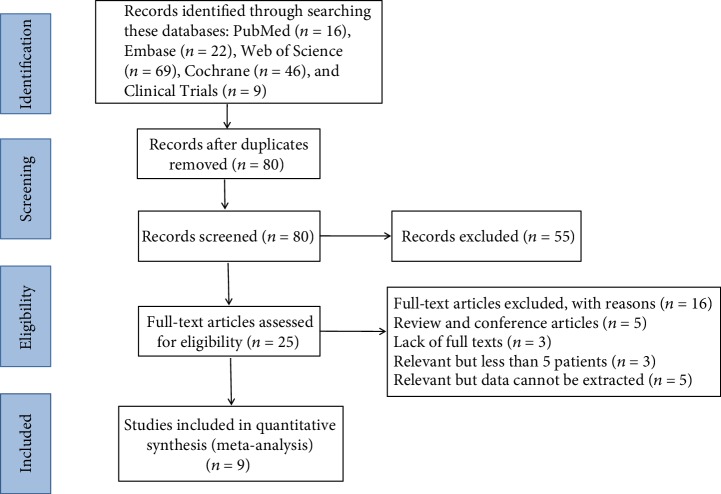
Flow diagram of the study selection process.

The search strategy retrieved 16, 22, 69, 46, and 9 studies from the PubMed, Embase, Web of Science, Cochrane, and Clinical Trials databases, respectively. A total of 82 duplicated studies were excluded, and the titles and abstracts of the remaining articles were screened by two reviewers. Subsequently, 25 studies with full text were evaluated. Among these studies, 3 were excluded due to less than 5 cases, 3 due to lack of full texts, 5 due to being review and conference articles, and 5 due to relevant data not being extractable. Finally, in this meta-analysis, 9 studies [10–18] on aMSCs for the treatment of MS were analyzed which included 133 transplanted patients (Figure 1).

### 3.2. Study Characteristic

The included 9 studies, along with the basic characteristics and sample sizes, are summarized in Table 1. Nine studies were all reported in English. Eight out of 9 studies were open-label uncontrolled studies and 1 was a randomized study. The MSCs in the eight studies were derived from the bone marrow, whereas one study was from adipose. Five studies used intrathecal injection for cell transplantation, and a mean number for every patient was less than 50 × 10^6^ cells. Three studies adopted intravenous injection with cell dosages of 1‐4 × 10^6^ MSCs/kg, while only one study included both methods. The follow-up period ranged from 6 to 96 months. For reporting the outcomes, all included studies reported the change of EDSS and magnetic resonance imaging (MRI), and they also reported some mild adverse effects.

### 3.3. Safety: Transplant-Related Mortality and Overall Mortality

Nine studies containing 133 cases reported common adverse effects which included transient low-grade fever, slight headache, backache, nausea/vomiting, iatrogenic meningitis, and urinary/respiratory infection. However, the results showed that no transplant-related deaths were observed during follow-up. There were 2 deaths in the overall studies, which occurred 8 and 40 months after completing the study (one due to severe spastic quadriplegia, the other by choking on food) [16]. The OM was 1% (95% confidence interval (CI) 0%–4%), without heterogeneity among studies (I^2^ = 0%, *p* = 0.98).

### 3.4. Efficacy: Rate of Disease Progression

**Figure 2:**
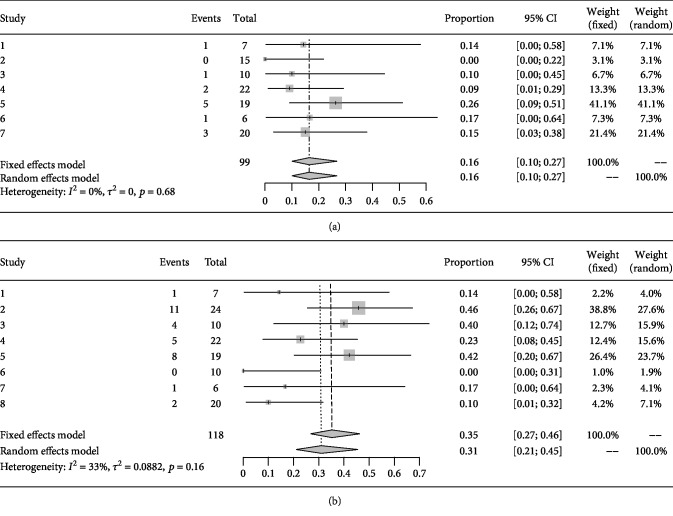
(a) Forest plot for 6 months progression rate in each study and pooled estimates. (b) Forest plot for 1 year progression rate in each study and pooled estimates.

After MSC transplantation, seven studies containing 99 cases reported the change of EDSS at 6 months [10–13, 15, 17, 18], while 118 cases in eight studies were observed at 1 year [10, 12–18]. The rates of disease progression were 16% at 6 months (95% CI 10%–27%, I^2^ = 0%, *p* = 0.68) and 35% (95% CI16.3%–31.8%, heterogeneity I^2^ = 33%, *p* = 0.16) at 1 year (Figures 2(a) and 2(b)).

### 3.5. Efficacy: Proportion of NEDA

**Figure 3:**
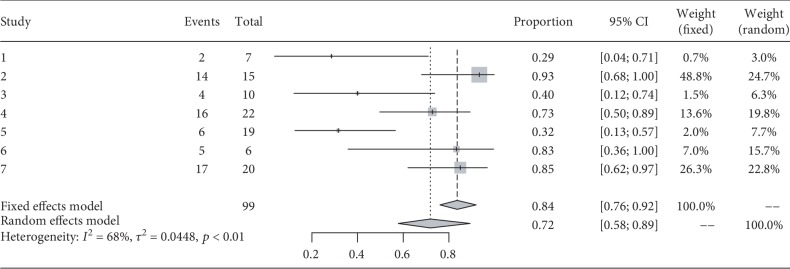
Forest plot for proportion of patients with no evidence of disease activity (NEDA) at 6 months.

**Figure 4:**
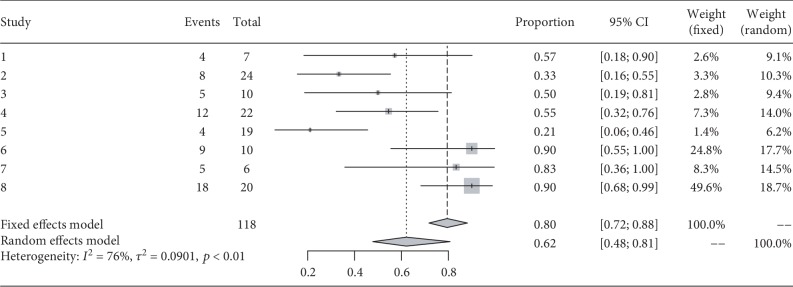
Forest plot for proportion of patients with no evidence of disease activity (NEDA) at 1 year.

During the follow-up, 99 patients were reported as having reached NEDA in seven studies. The percentage of NEDA patients was 72% at 6 months (95% CI 58%–89%, I^2^ = 68%, *p* < 0.01). One year following transplantation, the data for 118 patients was derived from eight studies, and the percentage of NEDA patients was 62% (95% (CI) 42%–81%, I^2^ = 76%, *p* < 0.01) at 1 year (Figures 3 and 4).

### 3.6. Subgroup Analysis

**Figure 5:**
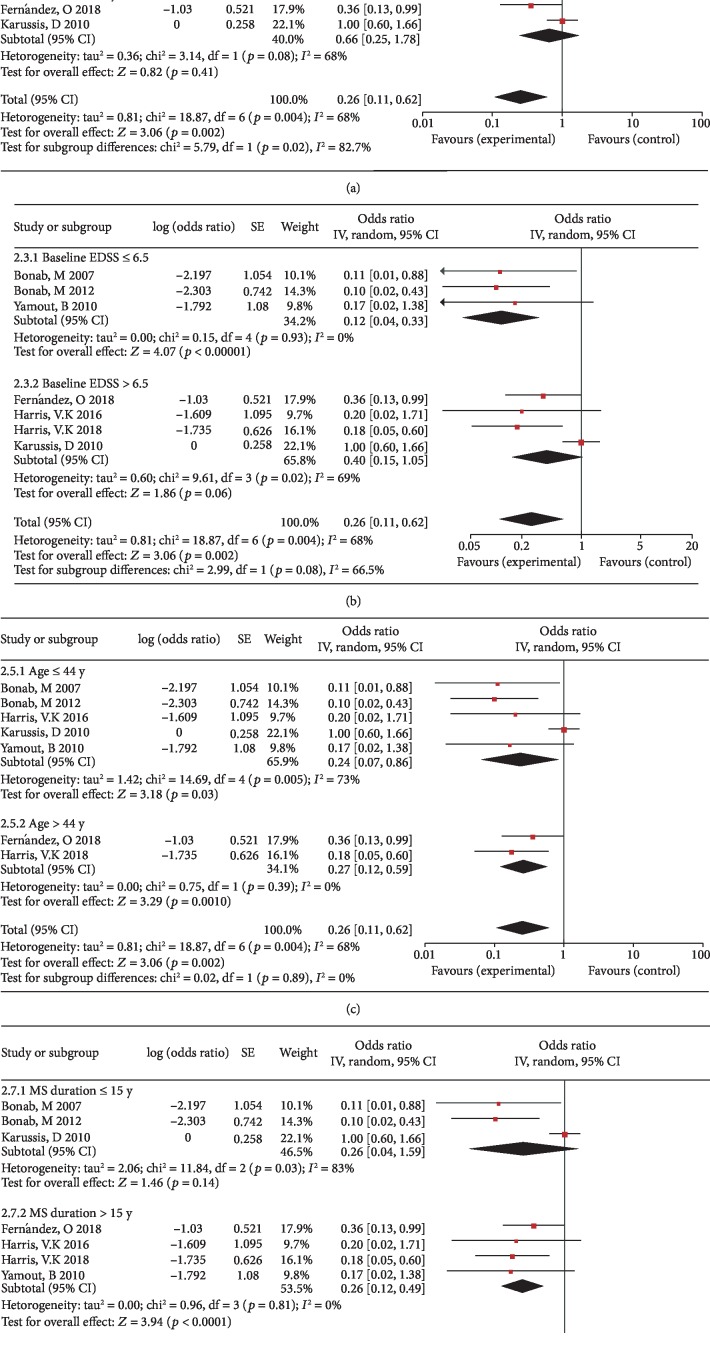
Forest plot of subgroup meta-analysis of the rates of disease progression at 6 months. (a) Subgroup analyses of intrathecal injection versus intravenous injection. (b) Subgroup analyses of baseline EDSS ≤ 6.5 versus baseline EDSS > 6.5. (c) Subgroup analyses of baseline age ≤ 44 y versus baseline age > 44 y. (d) Subgroup analyses of MS duration ≤ 15 y versus MS duration > 15 y. Squares indicate the risk ratio, and horizontal lines represent 95% confidence intervals.

**Figure 6:**
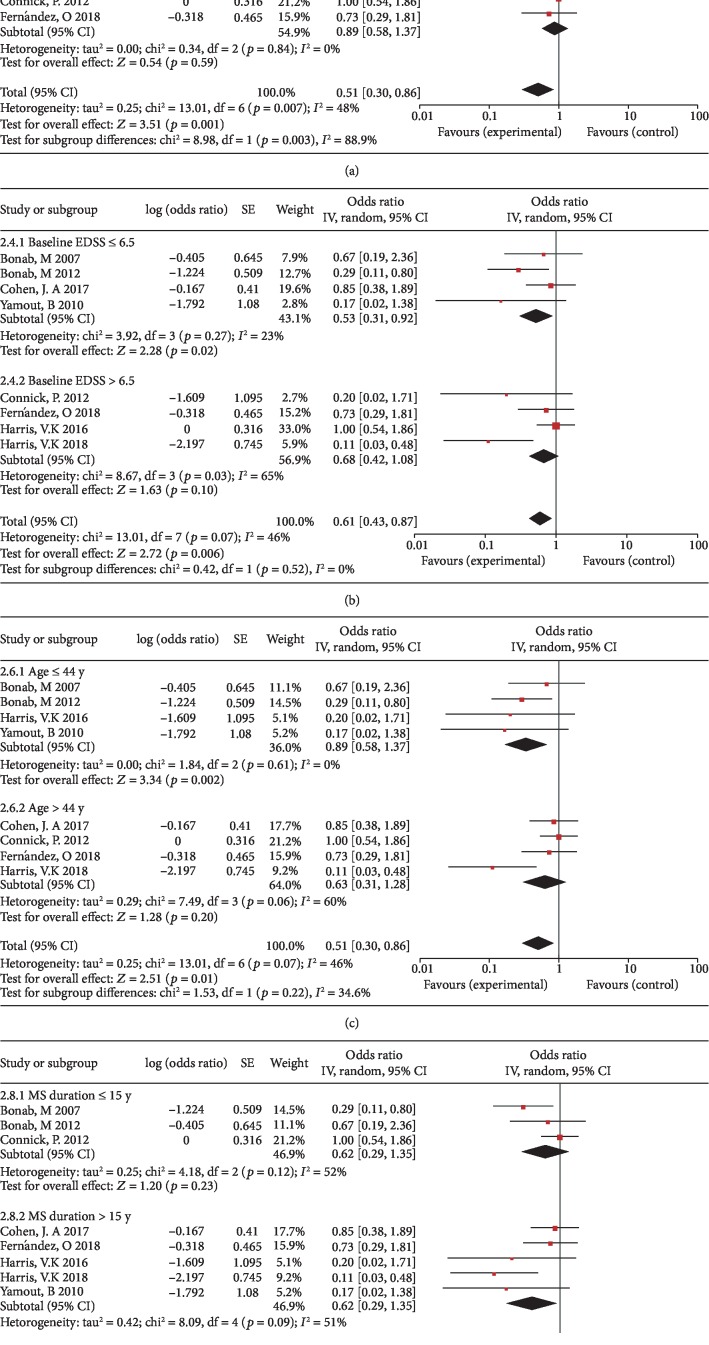
Forest plot of subgroup meta-analysis of the rates of disease progression at 1 year. (a) Subgroup analyses of intrathecal injection versus intravenous injection. (b) Subgroup analyses of baseline EDSS ≤ 6.5 versus baseline EDSS > 6.5. (c) Subgroup analyses of baseline age ≤ 44 y versus baseline age > 44 y. (d) Subgroup analyses of MS duration ≤ 15 y versus MS duration > 15 y. Squares indicate the risk ratio, and horizontal lines represent 95% confidence intervals.

We performed subgroup analysis based on age, EDSS, MS duration, and transplantation method. The subgroup analysis of disease progression rates at 6 months indicates intrathecal injection was more beneficial than intravenous injection on the disability progression rates (*p* = 0.02, I^2^ = 82.7%). However, there was no significant difference between the age, EDSS, and MS duration of different groups (Figures 5(a)–5(d)). The subgroup analysis of disease progression rates at 1 year also indicates intrathecal injection was more beneficial than intravenous injection on the disability progression rates (*p* = 0.003, I^2^ = 88.9%). There were no significant differences between the age, EDSS, and MS duration of the different groups (Figures 6(a)–6(d)). We performed subgroup analysis on the percentage of patients with NEDA at 6 months and 1 year, and the results showed that there were no significant differences between the age, EDSS, MS duration, and transplantation method of the different groups (data not shown).

### 3.7. Publication Bias

The assessment of publication bias cannot be conducted due to the number of included studies being less than 10.

## 4. Discussion

In recent years, the increase in reported MS patients treated with aMSC transplantation has led to debates about this therapeutic approach as a progressive MS treatment [19]. In this meta-analysis, we hope to provide useful information based on published articles. Only one of the included studies was a randomized study, and the rest were open-label uncontrolled studies to assess the role of aMSCs in different selected and treated MS patients. Therefore, it is very difficult to extract accurate information about the treatment of MS by aMSC transplantation, especially to understand its efficacy compared with other approved therapies for MS patients.

In this meta-analysis, we used different software to analyze the efficacy and safety of aMSCs for MS. However, there are some limitations: (1) the studies used different MSC types and sources which included adipose tissue and bone marrow, and the MSC transplantation procedure was not uniform in each study; (2) the characteristics of MS patients in each study were different: the majority of patients in the study were SPMS patients and the degree of disability was relatively advanced. Some studies even only included SPMS patients, and this type is highly likely to continue to progress after MSC transplantation; (3) the doses and transplantation methods of MSCs were different in each study; (4) this study included only 9 studies and 133 patients and was unable to perform some important subgroup analyses, such as different disease subtypes and cell sources; (5) the EDSS in assessing disease progression was significantly subjective, had poor reproducibility and low consistency, and it is difficult to compare and combine disease progression in different states [20, 21].

In this meta-analysis, we chose to use the rate of disease progression and percentage of NEDA as the main efficacy endpoints. The rate of disease progression was 16% at 6 months, while the rate reached 35% at 1 year. However, the 2-year disease progression rate was only 17.1% in the meta-analysis of autologous hematopoietic stem cell transplantation (aHSCT) for MS, and the 5-year disease progression rate was only 23.3% [9]. Considering that the above results may be related to the proportion of SPMS patients included in the study, since the study of aMSC transplantation in the treatment of MS mainly included SPMS patients, the proportion was mostly higher than 50%, and several studies even reached 90%-100%. Moreover, in the meta-analysis of aHSCT-treated MS patients, it was found that the maximal benefit profile was obtained in MS patients with relapsing-remitting stage [9]. It is not easy to compare the treatment outcomes of aMSCs with those reported drugs for MS in clinical trials, since the majority of patients enrolled in receiving aMSC transplantation generally have much higher EDSS scores and more aggressive stages of MS than the ones accepting disease-modifying drugs (DMDs). Among approved DMDs for MS, especially for SPMS, there has yet to be identified the most effective drugs to prevent disease progression [22]. The rate of progression after 6 months and 1 year was less than 10% and 20%, respectively, in SPMS patients treated with siponimod [23]. At the same time, in the clinical trial on the effect of natalizumab on disease progression in SPMS patients, the rate of progression after 2 years was 16% [24]. It is worth noting that the EDSS at enrollment for the two studies was 3.0-6.5, while the median EDSS is higher than 6 in this meta-analysis [23, 24]. Despite this, the results of this meta-analysis are robust, and the efficacy of aMSC transplantation for MS should be interpreted cautiously due to the limited studies and lack of control data.

In terms of safety, no transplant-related deaths were observed in all included studies. However, in the meta-analysis of aHSCT for MS, the pooled estimate of transplant-related mortality (TRM) was 2.1% [9]. There were 2 deaths in the overall studies during the follow-up, one due to severe spastic quadriplegia and the other due to choking on food [16]. In addition, there were some adverse effects reported in the enrolled studies which displayed as temporary and light side effects, including fever, slight headache, backache, nausea/vomiting, iatrogenic meningitis, and urinary/respiratory infection. All in all, it is safe to treat MS with aMSC transplantation.

In fact, subgroup analysis shows that the rate of disability progression of aMSC transplantation with intrathecal injection was less than that with intravenous injection both at 6 months and 1 year, and there was statistical difference (*p* < 0.05). In this analysis, the 6-month disease progression rate for EDSS ≤ 6.5 is less than EDSS > 6.5 and the 1-year disease progression rate for age ≤ 44 y is also less than age > 44 y, unfortunately there were no statistical differences (*p* > 0.05). Therefore, the largest benefit profile of aMSC transplantation for MS can be obtained in patients with aMSC intrathecal injection. Some data also demonstrates that intraperitoneal injection of adipose MSCs in EAE resulted in higher Treg cells expression and IL-4 production compared with intravenous route [25].

Animal studies on MS have shown that MSCs suppressed pathogenic effector CD4^+^ T cells, increased numbers of Tregs, and modulated effector CD8^+^ T cell subsets [6, 26]. In addition, MSCs exert a protective role in EAE by modifying microglia cells, including increasing the M2 and decreasing the M1 phenotype [8]. Furthermore, it should be noted that MSCs can be obtained from many human tissues, especially adipose tissue, which includes a majority of stem cells. Therefore, although this meta-analysis on MSCs in the treatment of MS has a relatively high rate of disability progression compared to other treatment regimens, we still have reason to expect the efficacy of MSC.

In this meta-analysis, we demonstrate the safety of aMSCs for the treatment of MS patients. However, the efficacy of aMSC transplantation in MS should be interpreted cautiously compared with those reported treatment regimes, and more random clinical trials are needed to clarify the efficacy of MSCs for treating MS. This meta-analysis also shows the significant association of aMSC intrathecal injection with lower disability progression rate. All in all, comprehensive consideration of results indicate that aMSC transplantation is safe, and to better evaluate the efficacy more studies need to be investigated in the future.

## 5. Conclusions

Cell transplantation with aMSCs for treatment of MS is safe, with a largest benefit profile being obtained in patients with aMSC intrathecal injection.

## Figures and Tables

**Table 1:** Basic demographics and clinical characteristics of each included study.

Authors	Sample size, *n*	Follow-up, month	Age, year	EDSS	MS subtype, %	MS duration, year	Cell source	Transplantation way
Bonab et al.	10	19 (13-26)	33 (22-40)	5.15 (3.5-6)	SPMS (80%) PPMS (20%)	11.2 (3-21)	Bone marrow	Intrathecal injection

Karussis et al.	15	6	35.3 ± 8.6	6.7 (4-8)	NA	10.7 (5-15)	Bone marrow	Intrathecal and intravenous injection

Yamout et al.	7	12	42 (34-49)	6.5 (4.5-7.5)	SPMS (100%)	19.9 (11-39)	Bone marrow	Intrathecal injection

Bonab et al.	22	12	35.2 (23-50)	6.2 (5.5-7)	SPMS (91%) PRMS (9%)	8.68 (5-14)	Bone marrow	Intrathecal injection

Connick et al.	10	12	48.8 (40–53)	6.1 (5.5–6.5)	SPMS (100%)	14.4 (5–26)	Bone marrow	Intravenous injection

Harris et al.	6	88.8 (48-96)	43 (28-64)	7.3 (6.5-9)	SPMS (67%) PPMS (33%)	17 (7-27)	Bone marrow	Intrathecal injection

Cohen et al.	6	12	46.4 ± 5.2	6 (3–6.5)	SPMS (58%) RRMS (42%)	15.4 ± 9	Bone marrow	Intravenous injection

Fernández et al.	19	12	46.3 ± 8.85	7.64 ± 0.575	SPMS (100%)	17.05 ± 7.4	Adipose	Intravenous injection

Harris et al.	20	12	49 (27-65)	6.8 (3.5-8.5)	SPMS (80%) PPMS (20%)	18.8 (10-32)	Bone marrow	Intrathecal injection
